# Revised french military transfusion doctrine for large-scale combat operations: a consensus framework

**DOI:** 10.1186/s13054-026-05862-9

**Published:** 2026-04-10

**Authors:** Sandrine Pons, Benoit Frattini, Yann Daniel, Clément Derkenne, Marine Chueca, Michael Cardinale, Nicolas Libert, Julien Bordes, Éric Meaudre

**Affiliations:** 1 Blood bank, Haemovigilance and Biology Department, Sainte Anne Military Training Hospital, Toulon, France; 2 Emergency Medicine Department, Paris Fire Brigade, Paris, France; 3 French Military Health Service, Paris, France; 4 French Military Blood Institute, Clamart, France; 5 Intensive Care Unit, Sainte Anne Military Training Hospital, Toulon, France; 6 Ecole du Val-de-Grâce, French Military Health Academy, Paris, France; 7 Intensive Care Unit, Percy Military Training Hospital, Clamart, France

**Keywords:** Whole blood, Transfusion doctrine, Military medicine, Haemorrhagic shock, Large-scale combat operations, Low intensity conflicts, Operational medicine, French armed forces, Haemovigilance, Trauma resuscitation

## Abstract

**Background:**

Large-scale combat operations (LSCO) pose unique logistical and clinical challenges to haemorrhagic casualty management. The existing French military transfusion doctrine, originally designed for low-intensity conflicts (LIC), does not meet the demands of LSCO. This work aimed to develop a revised, consensus-based transfusion doctrine integrating operational, medical, and safety requirements for LSCO environments.

**Methods:**

At the request of the Central Directorate of the Military Health Service, a Delphi-based consensus process was conducted between November 2024 and April 2025. Eight senior military physicians—anaesthesiologist-intensivists, emergency and operational clinicians, and medical biologists with over five years of specialty experience—reviewed eleven thematic domains identified for revision. Drafts, developed from doctrinal texts, operational feedback, and international literature (including systematic reviews), were iteratively refined through three Delphi cycles until full consensus (100%) was reached. This consensus reflects expert doctrinal guidance for LSCO operational preparedness under limited/heterogeneous evidence rather than proof of clinical efficacy. The resulting doctrine was compared with the previous LIC framework and international LSCO transfusion strategies.

**Results:**

The new doctrine establishes a scalable, safety-focused system integrating forward whole-blood resuscitation, enhanced logistical autonomy, and reinforced haemovigilance. Cold-stored whole blood will be prioritised as the primary resuscitation product for Roles 1 and 2, while Role 3 will employ component therapy. A new dedicated operational medical unit will oversee in-theatre collection, testing, and distribution. Forward transfusion by trained nurses using universal blood products under predefined criteria will be authorised, with strict traceability and targeted training ensuring transfusion safety in LSCO.

**Conclusions:**

This consensus defines the modern French transfusion doctrine for LSCO, combining operational flexibility with rigorous safety oversight and alignment with international standards.

## Introduction

The war in Ukraine and the current geopolitical context are prompting European countries to prepare for the possibility of large-scale combat operations (LSCO). LSCO are defined as “a very violent and sustained confrontation between forces, in all fields and environments, which may be localised and sporadic or sustained” according to the joint operational terminology glossary. Under LSCO conditions, logistical and tactical constraints impose a dramatic change in the scale of healthcare support: casualty numbers may surge beyond established capacities, with peaks that can overwhelm the system. Whether in low-intensity conflicts (LIC) or in LSCO, the ultimate objective of the medical support is to deliver optimal care from the frontline to evacuation back to metropolitan France. However, modern anti-aircraft threats can prevent or delay aerial medical evacuation, thereby increasing transit and evacuation times to the nearest operational medical unit (OMU) including surgical facilities, which may be far from the front line (i.e. ≥ 10 km).

The concept of the “Golden Hour” remains central in combat casualty care: minimising time to effective haemorrhage control and definitive care is critical. Under LSCO conditions, early blood-based resuscitation, including transfusion, when available, should be understood primarily as stabilising time-buying measures until definitive haemorrhage control can be achieved, but it cannot replace surgical or procedural haemorrhage control when required, which remains in those cases the cornerstone of survival.

Haemorrhage is a leading cause of death in combat and a major contributor to potentially survivable fatalities. It represents a substantial proportion of early deaths and remains a key focus of combat casualty care, including rapid haemorrhage control and blood-based resuscitation where available.

It accounts for approximately 90% of battlefield fatalities and 80% of deaths in Role 2 medical facilities [1].

In combat trauma with suspected life-threatening haemorrhage, early blood-based resuscitation and balanced transfusion strategies have been associated with improved outcomes in several observational series [2, 3]. However, these data are vulnerable to survivor bias and confounding, as plasma is often available later than RBCs and early deaths may be underrepresented or may reflect injury patterns not modifiable by transfusion strategy; time-dependent analyses have shown that the apparent survival benefit of high ratios can be attenuated when exposures are modelled as time-varying [4]. Whole blood–based resuscitation has also been reported as feasible in this context and has been associated with improved haemostatic resuscitation in some series, although high-quality comparative evidence remains limited [5, 6]. In LSCO, rising scale and tempo sharply will increase transfusion demand. The post-2020 resurgence of large-scale conflict compels a new doctrine for high-intensity, multi-domain operations. How do we meet greater quantitative and qualitative transfusion needs without compromising safety or logistics for civilian and military casualties?

## Methods

In October 2024, in anticipation of potential Large-Scale Combat Operations (LSCO), the Central Directorate of the French Armed Forces Health Service mandated a revision of the existing military transfusion doctrine to ensure operational readiness for high-intensity conflict. Given the doctrinal and operational nature of this task, a structured expert consensus process based on a Delphi-like methodology was selected.

A working group of eight senior military physicians was convened. Experts were selected according to predefined criteria: a minimum of five years of active-duty experience in their specialty, and demonstrated recognised expertise in transfusion medicine, trauma care, or operational medicine. The panel included anaesthesiologist–intensivists, emergency and operational medical unit clinicians, and medical biologist covering the full transfusion continuum, from frontline transfusion support to product qualification, haemovigilance, and training. Triage and rationing decisions during mass-casualty events are beyond the scope of this paper; our focus is the LSCO blood support architecture, to be implemented alongside established casualty triage and haemorrhage-control/damage-control pathways.

The group was chaired by the Head of the Chair of Anaesthesia and Intensive Care for the Military Health Service, who ensured representation across all critical stages of the transfusion chain.

The Delphi process unfolded over three iterative consultation cycles, corresponding to five structured meetings. Due to the accelerated preparation for a LSCO, the drafting process was carried out over a relatively short period conducted between November 2024 and April 2025. Eleven thematic domains were identified for revision by initial agreement of the panel. For each domain, two to three experts prepared draft proposals based on French doctrinal and regulatory texts, operational feedback from recent deployments, and a critical review of the international literature.

Importantly, the literature review included not only observational military studies but also randomised controlled trials and systematic reviews reporting neutral or negative findings regarding transfusion strategies, including prehospital blood transfusion and transfusion ratios. These data were explicitly discussed during the Delphi rounds and informed a cautious framing of recommendations.

Consensus thresholds were predefined as follows: strong consensus when ≥ 80% of experts agreed, and full consensus when 100% agreement was reached. Ultimately, all domains achieved full consensus. This agreement reflects operational feasibility, safety considerations, and doctrinal coherence under LSCO constraints, rather than evidence of clinical superiority of one transfusion strategy over another.

No formal methodologist or statistician participated in the panel. This is acknowledged as a limitation. The objective of the process was not to produce evidence-based clinical guidelines or perform quantitative synthesis, but to define a pragmatic, safety-oriented transfusion doctrine applicable to LSCO environments for the French Army.

Throughout the process, secure digital collaboration tools were used, allowing continuous remote interaction and document version control within a restricted-access workspace.

The resulting doctrine, validated after three discussion rounds of Delphi cycle for consensus, was structured into six chapters: transfusion rationale, blood components, operational preparation, transfusion organisation, supply chain, and training. The final version was presented in April 2025 and authorised for dissemination by the Operational Headquarters of the Military Health Service. In this manuscript, LSCO-specific requirements are redefined, and a comparative analysis with the prior LIC framework is provided, encompassing pre-deployment preparation, in-theatre adaptation, and post-transfusion haemovigilance. While grounded in standards aligned with peacetime haemovigilance, the revised doctrine explicitly incorporates LSCO contingency pathways (‘exception’ procedures) governed by predefined risk management to allow graded adaptation when evacuation, resupply, or staffing are contested. Indeed, even though the peacetime framework is the baseline for preparedness, LSCO conditions may require adapted policies, exceptional-situation procedures and crisis-specific derogations, consistent with national command and haemovigilance requirements included in the French framework.

## Results

### Increased transfusion demand in LSCO

#### Quantitative demand

Under LIC conditions, the transfusion requirement remained limited. Between 2013 and 2020, French overseas missions in sub-Saharan Africa administered on average 225 blood components (BC) per year, totalling 1,795 units, predominantly red blood cell concentrates (RBC) (58%), lyophilised plasma (FLyP) (30%) and a small proportion of warm-fresh whole blood (WF-WB)(12%) [7]. In 2021, Cold-stored whole blood (CS-WB) was sent for the first time for overseas missions, in small quantity and mainly for special forces and evacuation teams. Under such circumstances, supply strategies were primarily driven by product shelf life and occasional urgent resupply.

In contrast, LSCO scenarios will imply a marked escalation in transfusion demand. To illustrate potential orders of magnitude, we modelled a hypothetical scenario of 100 casualties per day managed by the French Military Health Service within a coalition framework. Based on historical reports from World War II, the Korean War, and more recent conflicts, published estimates suggest that approximately 15–25% of combat casualties may require transfusion [1, 7, 8]. For planning purposes, we conservatively assumed a transfusion rate of 20%.

The expected volume of transfusion per casualty remains highly variable and dependent on injury patterns, evacuation delays, and access to surgical haemorrhage control. For logistical planning, we assumed a requirement of up to eight units of whole blood or whole-blood equivalent per transfused patient, in line with previously published military blood planning factors [8]. Under these assumptions, daily requirements could reach approximately 160 whole-blood–equivalent units.

These estimates are not intended to predict actual clinical consumption but to illustrate the scale of logistical stress imposed by LSCO. Under such conditions, annual transfusion volumes would exceed those observed during LIC by several-fold, rapidly surpassing peacetime production capacities.

#### Qualitative demand

Under LSCO conditions, qualitative transfusion requirements will extend beyond volume alone. The prioritisation of cold-stored whole blood (CS-WB) is driven by operational considerations, including simplified logistics, reduced risk of transfusion errors, and rapid availability at forward echelons [5, 6, 8, 9]. Indeed, in trauma resuscitation, whole blood (WB) has been increasingly adopted in some military and civilian settings primarily as a pragmatic approach to hemostatic resuscitation and for its logistical simplicity as you can potentially deliver red cells, plasma, and platelets in a single product. This balanced haemostatic product in a single unit may be advantageous when laboratory support and product diversity are limited.

However, comparative clinical evidence versus balanced component therapy remains limited and heterogeneous. Available studies suggesting improved outcomes with whole blood are predominantly observational, subject to significant bias and cannot establish a clear mortality benefit. Randomised controlled trials directly comparing CS-WB with component-based resuscitation are lacking. As such, the prioritisation of whole blood within the French LSCO doctrine reflects operational feasibility and safety considerations, rather than proven clinical superiority.

Since 2021, in French military operations, group O and A RBC stocks in emergency settings have been supplemented by CS-WB (leukocyte-depleted, group O Rh:D, with low anti-A/B titre) [10]. Its preparation requires specific collection kits allowing both leukodepletion and platelet preservation. In situations of equipment shortage, available kits may be used. These may either lack leukodepletion to prioritise rapid availability and the presence of all blood components, including platelets, or include leukodepletion filters without platelet preservation. The choice of kit will be made in order to meet operational demands and scheduling constraints while ensuring the highest possible level of safety. CS-WB use is limited to RhD positive units because in life-threatening haemorrhagic emergencies, the imperative is rapid resuscitation; alloimmunisation risk is managed later in care [11]. The risk of foetal loss due to anti-D alloimmunisation in RhD negative women of childbearing potential receiving D-positive RBC in emergency settings has been shown to be very low [12].

Under LSCO conditions, the objective will be to supply Role 1 and Role 2 medical units with so-called universal blood products, ensuring immediate transfusion capability close to the front line. Role 3 facilities will not be prioritised for these universal components, as they will have access to the full range of conventional blood components required for comprehensive casualty management.

Collection of warm fresh whole blood (WF-WB), under regulatory procedures, remains permissible but will be operationally more challenging. Where WF-WB is collected as a last-resort bridging strategy, procedures prioritise speed and safety essentials (donor identification, rapid eligibility checks, rapid testing and traceability). Leukoreduction is a characteristic of centrally prepared products and is not included in the WF-WB collection procedure, as it would delay transfusion.

In the absence of CS-WB supply, pre-hospital FLyP transfusion will become crucial, particularly when delays are prolonged [13]. The use of platelet concentrates will also be desirable, ideally stored at 4 °C with extended shelf life.

In exceptional circumstances where French blood products are unavailable, recourse to allied nations’ blood products will occur only as a last resort and will follow the priority order defined by NATO Standardisation Agreement (STANAG): first by “type of blood product” (Category 1, then 2, and finally 3), and second by “type of Operational Medical Unit,” with Role 3 facilities prioritised, followed by Role 2, and only ultimately Role 1.

### Organisational adaptations for LSCO

#### Pre-deployment preparation

Every deployed soldier is both a potential recipient and potential donor [14]. Operational transfusion preparation is thus mandatory pre-deployment: each soldier undergoes dual ABO/Rh typing and full qualification as a donor. This sample, analysed exclusively at the French Military Blood Institute (FMBI-HQ), comprises immuno-hematologic tests (e.g., anti-haemolysin screening) and full infectious disease screening (including, in particular, syphilis, HIV, HBV, HCV, HEV, HTLV). These data are transferred to each individual’s medical record, facilitating donor registers in the operations theatre.

#### On-site (Theatre) resources: FMBI-operational medical unit (FMBI-OMU)

In LIC scenarios, supply of BCs was managed from national territory via the FMBI-HQ, and forward blood banks of 10 to 30 units were held under the control of Role 2 anaesthesia teams. In LSCO, the increase in demand and the complexity of the supply chain will require deployment to theatre of a dedicated FMBI-OMU located in the Role 3 area. Its two primary missions will be: (1) reception, storage, and distribution of high-volume BC shipments from national territory to OMUs; (2) local collection and preparation of CS-WB to reinforce supply. The FMBI-OMU will aim to replicate national territory-level qualification procedures to meet NATO interoperability standards. The collection of WF-WB will remain the responsibility of Role 1/Role 2 units under specific conditions.

#### Transfusion in the operational theatre

In LSCO, periods of scarcity are foreseeable as situation is characterised by uncertainty, prolonged evacuation delays, and the potential for casualty numbers to exceed available medical and transfusion resources. The doctrine therefore requires predefined triage and allocation principles when casualties outnumber available units, prioritising patients with potentially survivable haemorrhage, achievable haemorrhage control within an operationally realistic time frame, and expected physiological benefit from temporising resuscitation. These principles are rooted in the military origin of medical triage, first formalised by Dominique-Jean Larrey during the Napoleonic Wars, to optimise the use of limited resources and maximise survival on the battlefield. When bleeding remains uncontrolled despite available measures and definitive control is not achievable in time, continued transfusion may be reassessed in favour of maximising lives saved [15]. These decisions are ethically complex and context-dependent, but unavoidable in LSCO scenarios.

##### Roles and responsibilities

Under LIC, the anaesthesiologist-intensivist or Role 1 physician handled prescription, transfusion, storage, traceability, and overall blood bank management. In LSCO, the FMBI-OMU will become the central node: it will receive, store, and allocate BCs to OMUs (Role 1, Role 2). At those levels, anaesthesiologist-intensivist or Role 1 doctors will then manage transfusion.

##### Distribution scheme

Under LIC conditions, FLyP was first-line at Role 1 and CS-WB could be used during evacuation; other BCs were mainly located in Role 2 or Role 3. In LSCO, the objective will be universal distribution of CS-WB across all OMUs, prioritised by feasibility constraints. The expected consumption across medico-surgical facilities is 20% before Role 2, 50% at Role 2, and 30% after Role 2 [7]. At Role 1, only universally compatible BCs (CS-WB O, RBC O, and FLyP) will be used to initiate transfusion until handover to higher care. At Role 2, CS-WB (O and A) will be prioritised; with additional ABO-compatible RBC and FLyP as needed. At Role 3, fresh frozen plasma (FFP) and platelet concentrates will be added to enable component-based transfusion guided by laboratory results. At all echelons, a safety buffer of FLyP will be maintained. Given LSCO constraints, WF-WB is excluded from regular inventory but remains an option. High-performance validated containers, and emerging logistical enablers (e.g., drones, robots), will be critical for mobile banking.

##### Adaptation of transfusion procedures

In degraded tactical situations—such as unit isolation, disruption of resupply, or mass casualty events—standard transfusion practices may require adaptation. While leukoreduction, strict shelf-life limits, and product discard policies represent peacetime safety standards, LSCO may necessitate context-driven risk–benefit assessments. The doctrine recognises that, in extreme circumstances, deviations from optimal storage or processing conditions may be considered when the alternative is certain death from haemorrhage. Specific procedures will be needed and the FMBI-OMU will be questioned whenever possible. For example, in LIC, there was already a shelf-life derogation for RBC from 42 to 49 days in case of necessity. For LCSO, there should be contingency inventory strategies prioritising products with favourable shelf-life/logistics, continuous temperature monitoring, and predefined rules for quarantine/discard when cold-chain integrity is uncertain. Visual inspection alone is insufficient to guarantee quality; therefore, any deviation from standard discard criteria—if authorised under exceptional circumstances—must follow a formal risk-managed pathway with documentation and haemovigilance under FMBI responsibility.

In LIC, transfusion was prescribed and supervised by physician, and often performed by nursing staff aware of the patient’s blood group. In LSCO, due to tactical constraints and extended evacuation times, the doctrine will allow for task delegation to trained non-physician personnel under mandatory predefined rationalised protocols, reflecting operational realities. At Role 1, a nurse—whether isolated or not—will be able, independently, to initiate transfusion of universally compatible BCs (CS-WB, RBC O and FLyP) if two of the following criteria are present: heart rate > 110/min, systolic blood pressure < 90 mmHg, haemoglobin < 11 g/dL, positive FAST examination, or penetrating trauma (criteria’s aligned with existing literature) [16, 17]. The priority order will remain WB first, RBC + FLyP (1:1) next, and single product if only one is available. For universal WB or O RBCs, the mandatory pre-transfusion ABO testing could be performed upon unit receipt rather than immediately before transfusion ("pre-tested" universal BCs), thereby allowing omission of recipient ABO typing in emergencies. Traceability will remain mandatory [18]. Finally, level 2 combat lifesavers — medically trained combat personnel (section medics or stretcher-bearer rescuers) certified by the Operational Health Training Centre — will be authorised to monitor a casualty receiving an ongoing blood transfusion. Training programs are being revised accordingly, with digital modules, serious games, and in-person refresher training developed by FMBI and the Armed Forces Health Academy. These protocols allowing, in isolated tactical scenarios, that transfusion initiation with prepositioned universal products might be performed by trained personnel according to protocol and monitored by certified combat medical personnel, are intended to bridge time to haemorrhage control. These measures are tightly framed within training, traceability, and haemovigilance requirements to minimise risk while preserving lifesaving capability. They must be coupled with the earliest feasible evacuation to a capability for surgical or procedural control. Adaptations in combat medicine are being developed to mitigate shortages of physicians/nurses in austere settings (e.g., competency-based task shifting supported by protocols and remote oversight). Authorisation to collect/administer blood remains governed by national regulations and is outside the scope of this work.

**Fig. 1:**
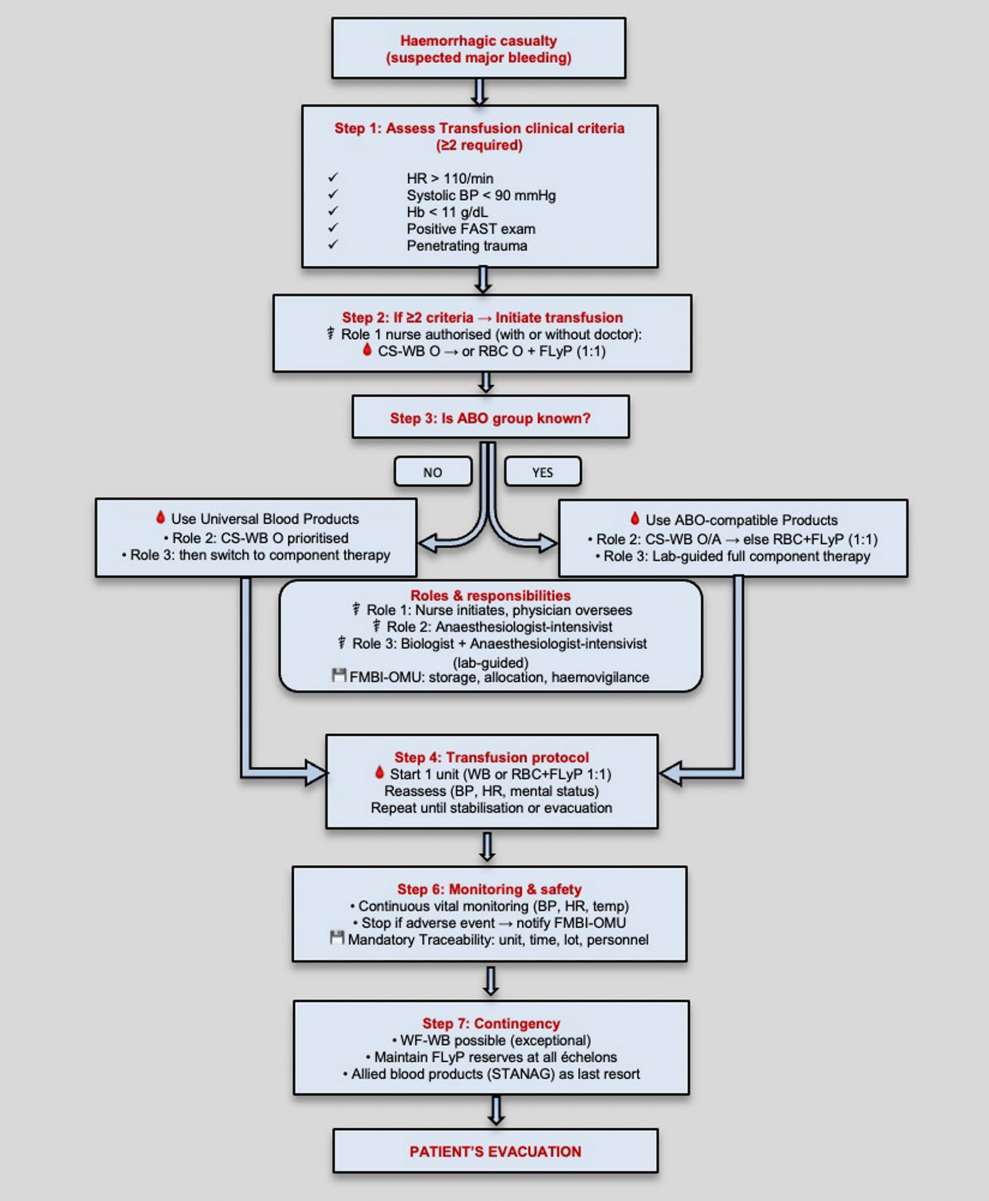
Structured decision pathway for haemorrhagic casualty management under LSCO. The algorithm integrates initiation criteria (≥ 2 clinical parameters), ABO-based product allocation, and role-specific responsibilities to ensure transfusion safety, traceability, and operational efficiency

This decentralised but standardised approach ensures continuity of care and transfusion safety from the point of injury to Role 3 facilities. A structured decision algorithm was subsequently developed to standardise transfusion practices under LSCO conditions, defining initiation criteria, product allocation according to ABO compatibility, and clearly delineated responsibilities from Role 1 to Role 3 (Fig. 1). This framework aims to ensure timely haemostatic resuscitation, optimise cold-stored whole blood utilisation, and maintain haemovigilance across the operational chain.

#### Role 4/Rear-echelon monitoring and haemovigilance

##### Haemovigilance and traceability

The FMBI director remains responsible for haemovigilance in the operation theatre. In LIC, in the operation theatre, his relays were the doctors in charge of OMUs (anaesthesiologist-intensivists or Role 1 doctors). In LSCO, the FMBI-OMU will provide transfusion advice on the operation theatre, ensure the traceability of BCs made available to OMUs, and monitor adverse events in donors or recipients related to the use of BCs collected and prepared on the national territory or operation theatre. For WF-WB collections, field tubes are sent to FMBI-HQ to permit post-hoc qualification and serology control. Furthermore, upstream, as part of the pre-deployment preparation, the performance of many pre deployment tests may reveal a significant number of previously unknown infections (HIV, HBV, etc.) and/or the presence of irregular agglutinins requiring medical treatment and raising questions about the suitability of staff for the mission.

Specific forms will be provided to doctors at selection centres to guide them in the event of seropositive test results or the positive detection of irregular agglutinins.

##### Blood group determination

All deployed personnel must carry a medical blood group card with dual ABO-RH 1 (D) and RH-KEL1 determinations, results must be recorded and digitised. At Role 4, when pre-transfusion samples are unavailable, previous history of repeated transfusions may render blood grouping indeterminate due to mixed red-cell populations. A standardised algorithm is required to interpret such cases, recover the patient’s native blood group, and minimise reliance on universal RBCs. Accordingly, an admission blood sample should be obtained as early as possible to preserve baseline immuno-haematologic and infectious-disease information.

## Discussion

### Evolution of the French transfusion doctrine: from LIC to LSCO

Since World War II, the French military transfusion doctrine has progressively balanced safety, availability, and operational deployability. The introduction of lyophilised plasma (FLyP) in 1949 was a milestone, enabling battlefield transfusion autonomy in remote theatres [19]. In the late twentieth century, the establishment of the French Military Blood Institute and the implementation of robust haemovigilance and traceability procedures constituted a paradigm shift toward harmonisation with European civilian standards, enhancing quality over mere supply volume [20].

Operational lessons from Afghanistan, the Sahel, and other deployments in the 2010 s catalysed a doctrinal move toward earlier transfusion—embodied by the “blood far forward” concept—aiming to reduce time to intervention and shift components forward in the casualty pathway [21]. Advances in FLyP, such as pathogen inactivation, preserved logistic advantages while increasing safety, reinforcing its continued role in austere settings [19]. Published reports indicate that prehospital use of lyophilised plasma in combat and expeditionary settings is feasible, and some observational datasets suggest a potential association with improved physiological or clinical outcomes; however, the overall certainty of evidence remains limited and context-dependent [4, 7, 22].

The persistence of haemorrhagic shock as the primary cause of preventable combat death led the French military to re-explore whole blood resuscitation. From 2017, a program of warm fresh whole blood (WF-WB) dedicated to Role 1 teams was deployed, followed by the adoption of low-titer group O whole blood (LTOWB) in forward zones (e.g., Sahel). These measures, aligned with allied practices, aimed to streamline logistics and enhance early resuscitation. A retrospective analysis over the first two years confirmed the practical feasibility and safety of LTOWB in operational environments [10].

Importantly, parts of the early observational literature in combat trauma evaluating high plasma ratios and early component delivery are susceptible to time-dependent and survivor biases, which may inflate apparent treatment effects [23]. Early deaths, occurring before blood products become available, were often excluded or underrepresented, leading to overestimation of apparent survival benefits. Subsequent analyses using time-dependent methods and evidence from randomised trials have produced more nuanced findings, such as apparent survival advantages reported in some early studies that are attenuated after adjustment for immortal time bias, underscoring the need for cautious interpretation [4]. Evidence supporting prehospital transfusion remains limited: the updated Cochrane review found little to no evidence of a difference between transfusion strategies for mortality overall, and reported no clear mortality difference for prehospital plasma versus standard care across trials, with evidence certainty varying by outcome [24].

Still, the French expert group has stated, in LSCO doctrine development, to interpret the available evidence as supportive of early balanced resuscitation principles and operational feasibility, while recognising that the magnitude of any survival benefit remains uncertain and may vary by setting, evacuation time, and haemorrhage control capability.

Within this context, the prioritisation of whole blood in LSCO should not be interpreted as evidence of clinical superiority over component therapy. Rather, it reflects operational considerations specific to high-intensity conflict, including simplified logistics, reduced cognitive load for frontline providers, minimisation of transfusion errors, and the ability to deliver a balanced haemostatic product when laboratory support and product diversity are limited.

The French doctrine therefore adopts a pragmatic position: whole blood is favoured when available and appropriate, but component-based transfusion remains essential, particularly at higher echelons of care where laboratory guidance, platelet concentrates, and surgical resources are accessible.

Today’s doctrine fuses multiple modalities—pathogen-inactivated FLyP, RBC, and warm-fresh or cold-stored WB—into a cohesive system, underpinned by unified haemovigilance, extended competencies for forward personnel, and a dedicated unit (FMBI-OMU) for theatre collection and distribution.

Under LSCO conditions, the feasibility of this system depends on scalable resource allocation**,** including increased personnel training and structuring of dedicated transfusion roles within operational units**.** Cold-chain constraints necessitate rapid adaptation of existing infrastructure and the development of modular storage and transport solutions to ensure product integrity across dispersed theatres. While cost implications remain significant—linked to equipment, training, and maintenance—the reuse of established logistical networks and progressive integration of innovative technologies (e.g., portable refrigeration, data-driven traceability, and pathogen-reduction platforms) mitigate these challenges. This transition represents a shift from a supply-centric to a forward-resilient, doctrine-driven system, capable of meeting LSCO demands without compromising safety or sustainability.

Table 1 provides a comparative overview of the key doctrinal, logistical, and operational adaptations required to transition from the LIC transfusion framework to the LSCO context.

**Table 1:**
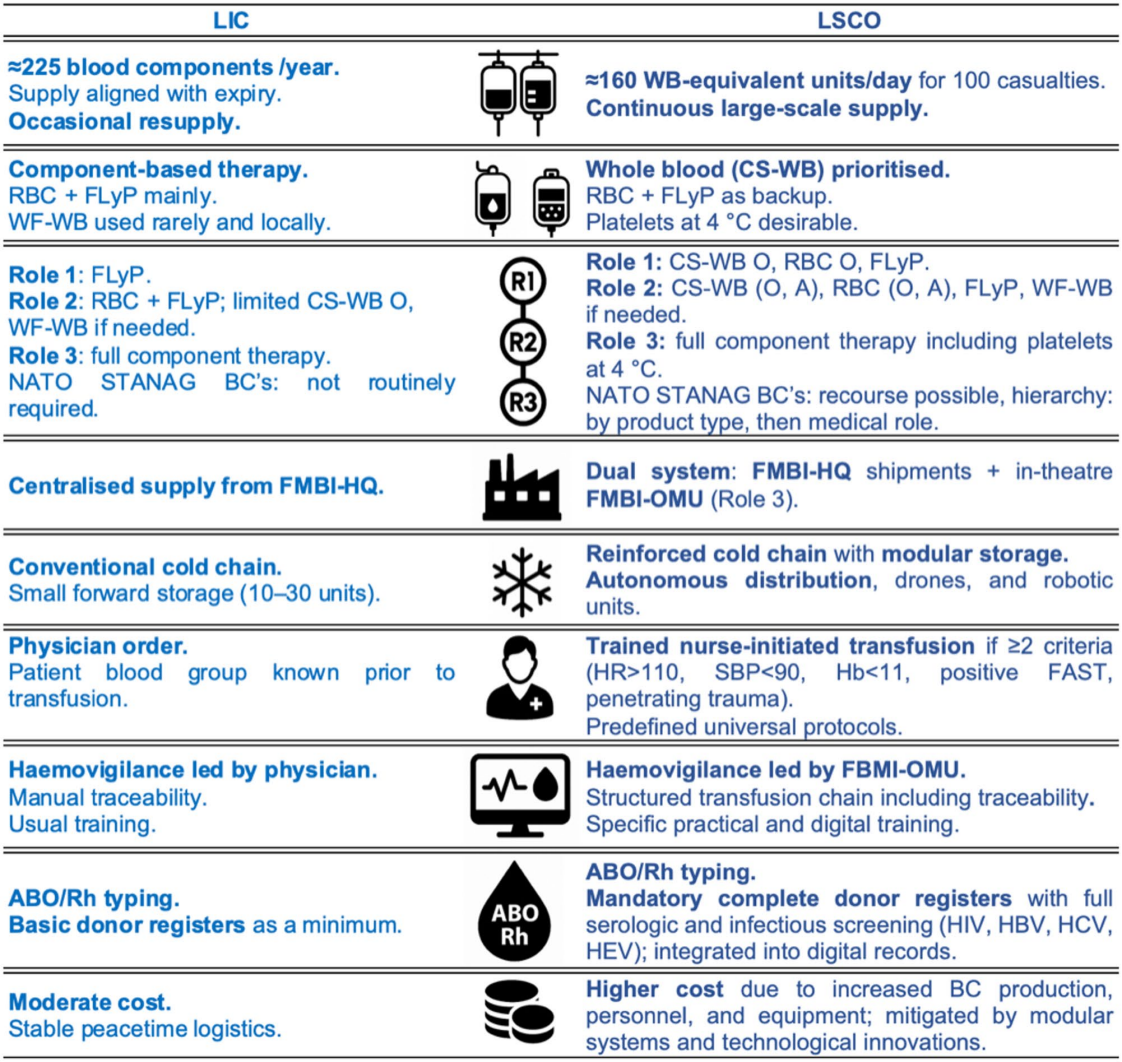
Evolution of the French military transfusion system from LIC to LSCO

### Civil–Military collaboration and strategic preparedness

Blood is both a civilian and military medical priority, requiring seamless coordination between clinicians, transfusion specialists, logisticians, and scientists. Across Europe, a shift toward a “whole-of-government” approach is underway, driven by lessons from the war in Ukraine. Civilian systems traditionally operate on a *just-in-time* model, whereas the military relies on *just-in-case* preparedness. Merging these philosophies is key to achieving resilience and sustaining capacity during large-scale crises.

Ensuring surge capability for LSCO demands shared planning, funding, and interoperable standards between civilian and military blood systems. European discussions have underscored the need for harmonised legislation, common stockpiling strategies, and standardised deployable products—such as reconstituted dried plasma—to strengthen collective readiness. Notably, the Directive 2002/98/EC of the European Parliament and of the Council of 27 January 2003 sets standards of quality and safety for the collection, testing, processing, storage and distribution of human blood and blood components, thereby providing the regulatory backbone for both civilian and defence transfusion systems [25].

On a theatre of operations, the necessary alignment between the transfused population and the local donor base is constrained by differences in ABO/Rh distribution—especially the scarcity of RhD-negative donors—so local stocks alone may not reliably meet demand. For planning purposes, typical distributions are: Europe—O totals ~ 32–55% (O + ~ 27–47%, O − ~ 4–9%), A ~ 31–49%, B ~ 8–24%; United States—O ~ 44% (O + 37.4%, O − 6.6%), A ~ 42%, B ~ 10%, AB ~ 4% [26]. This is particularly important because military medical facilities may treat civilian casualties, and civilian hospitals may treat NATO casualties from multiple member nations.

In civilian trauma care outside France, especially in the United States, the use of whole blood (WB) is increasingly documented. For instance, a Norwegian civilian helicopter emergency medical service (HEMS) implemented low-titer group O whole blood transfusion in major haemorrhage and reported feasibility and safety [27]. Other European studies and reviews highlight that WB transfusion is gaining traction in civilian trauma populations, with improved logistical simplicity and promising outcomes compared with component therapy [28, 29]. This illustrates an increasing civilian interest in whole-blood strategies, how military experience has influenced wider civilian haemostatic resuscitation practice and supports the argument for combined civil–military readiness, while acknowledging that the evidence base remains largely observational and context sensitive. Indeed, earlier military observational findings favoring higher plasma transfusion were vulnerable to survivor (immortal-time) bias and confounding, with time-dependent analyses attenuating the apparent survival effect. Randomised evidence has not confirmed large all-cause mortality reductions (e.g., PROPPR showed no significant difference in 24-h or 30-day mortality between 1:1:1 and 1:1:2) [23].”

Scaling blood support for LSCO raises legitimate concerns about civilian supply tension, donor burden, and the risk of inappropriate utilisation outside evidence-based indications. A resilient approach therefore requires explicit blood stewardship (clear triggers, audit, and feedback), pre-defined crisis allocation principles, and coordinated civil–military planning for surge production, stockpiling, and prioritisation. These safeguards must be balanced against transfusion-associated risks (infectious, immunologic, and logistic) and the operational imperative to preserve life while haemorrhage control is achieved.

The French approach, integrating the FMBI within a broader civil–military framework, exemplifies this evolving doctrine of mutual support and interoperability [30].

### International comparison: commonalities, divergences, and french specificities

#### Convergence of principles

Most LSCO-focused military doctrines converge toward three strategic goals: (1) bring the first transfusion as close as possible to the point-of-injury as possible; (2) provide balanced resuscitation that restores coagulation and oxygen-carrying capacity; (3) maintain haemovigilance and safety under austere conditions.

Nations differ in their product choices, sourcing models (prepositioned stock vs walking blood bank), and civil-military integration [31, 32]. The U.S. leads with mature LTOWB programs, integrated cold-stored WB use, formal walking blood bank (WBB) systems, and standardised protocols supported by the Joint Trauma System (JTS) Clinical Practice Guideline ID 21: “Whole Blood Transfusion” [33, 34]. The U.K., through the Defence Medical Services CGO “Major Haemorrhage Guideline”, emphasises dried plasma to mitigate cold-chain challenges while still enabling prehospital RBC/plasma transfusion [35, 36]. Israel has operationalised LTOWB in very forward units, with tight integration between tactical teams and evacuation chains [32, 37, 38]. Nordic countries (Norway, Sweden) adopt hybrid models combining prepositioned CS-WB and WBB protocols, exploiting civil–military interoperability [27, 39, 40]. Canada and Australia are formalising prehospital LTOWB/cold-WB policies in collaboration with civilian retrieval systems [41, 42]. Germany and Poland, with more fragmented emergency medical systems, are in earlier stages of adoption and integration [43, 44].

Table 2 provides a comparative overview of French and international transfusion support in LSCO.

**Table 2:**
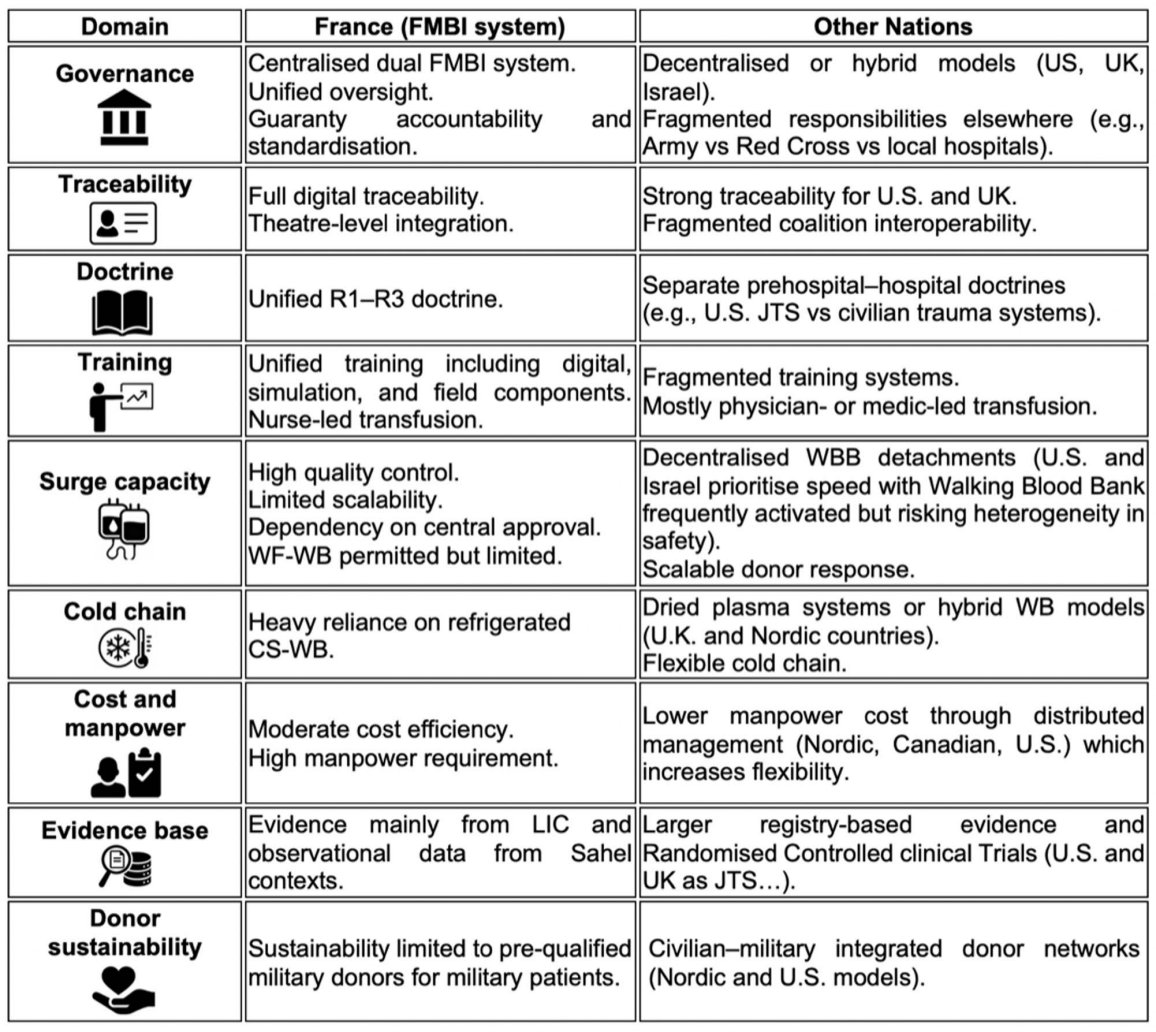
Comparative overview of French and international transfusion support in LSCO

From this, two broad paradigms emerge:

- Pre-deployed inventory (LTOWB/CS-WB/FLyP)**:** immediate availability at cost of cold-chain logistics (except for FLyP).
- Walking blood bank (WBB)**:** greater self-sufficiency and scalability, but requires strict donor screening, documentation, and post hoc quality controls [40, 45].

### French Doctrine: strengths, limitations, and perspectives

#### Strengths

The French LSCO transfusion doctrine combines alignment with international trends toward early whole-blood resuscitation and distinctive structural advantages. A centralised military-controlled network, through the French Military Blood Institute (FMBI), ensures unified governance of production, distribution, and haemovigilance, enabling high-quality standards and rapid decision-making. Doctrinal coherence across the evacuation chain—from forward units to hospitals—maintains interoperability and continuity of care even under LSCO conditions. A robust regulatory and training framework, integrating simulation, digital, and in-person modalities, standardises transfusion practice and enhances procedural safety. Finally, the system achieves a balance between responsiveness and safety through controlled forward transfusion protocols, central haemovigilance, and scalable logistics, providing both flexibility and resilience for LSCO operations.

#### Limitations

Despite these strengths, the French model remains exposed to several structural and scientific challenges. Dried plasma provides a partial solution to plasma logistics but cannot substitute for red cells or whole blood in oxygen delivery. The approach depends on robust cold-chain maintenance, reliable donor availability, and real-time registry updates—all of which represent potential vulnerabilities in contested environments. Moreover, implementation requires extensive and sustained training, both technical and doctrinal, for all categories of deployed personnel.

#### Perspectives

From a scientific standpoint, current evidence supporting whole-blood resuscitation in military and civilian trauma remains largely observational. Systematic multicentre registries and operational trials are critically needed to evaluate transfusion strategies and generate normative LSCO-specific guidelines [10, 46, 47]. Future research should ideally include randomised controlled trials comparing cold-stored whole blood (CS-WB) with red-blood-cell plus fresh-frozen plasma (RBC + FFP) resuscitation in civilian trauma settings, and the establishment of a dedicated LSCO registry would enable systematic evaluation of outcomes, safety data, transfusion practices and resource utilisation, informing future doctrinal refinements.

In sum, while French doctrine converges with allied trends toward early WB use, its distinctive strength lies in its centralised governance, doctrinal unity, and built-in safety structures, attributes that may offer valuable models for coalition interoperability and future NATO harmonisation.

## Conclusion

Large-Scale Combat Operations impose tactical, logistical, and medical constraints that will necessitate a fundamental shift in transfusion doctrine compared to LIC. Whole blood, preferably leukocyte-depleted, platelet-rich, and ideally ABO-compatible, is the preferred resuscitation fluid. In theatre, a dedicated FMBI-OMU will handle reception, distribution, and advice, as well as on-site preparation of CS-WB from qualified military donors. Forward medical personnel will apply standardised criteria to initiate transfusion, supported by rigorous traceability and haemovigilance. This reorganisation will raise the level and continuity of care across all stages of the casualty pathway, enhancing survival potential before surgical interventions. Implementation of such a doctrine demands regulatory adaptation, sustained training, and resource investment to uphold safety under the pressures of large-scale combat operations.

## Data Availability

Not applicable. No datasets were generated or analysed for this review. All information discussed is available in the published literature cited in the References.

## Abbreviations

BC: Blood components.
CS-WB: Cold-stored whole blood.
FFP: Frozen fresh plasma.
FLyP: French lyophilised plasma.
FMBI-OMU: French military blood institute- operational medical unit.
FMBI-HQ: French military blood institute headquarters in Clamart.
LSCO: Large-scale combat operations
LIC: Low-intensity conflicts.
OMU: Operational medical unit.
PC: Platelets concentrate.
RBC: Red blood cell concentrates.
WB: Whole blood.
WF-WB: Warm-fresh whole blood
